# *Plasmodium* manipulates the expression of host long non-coding RNA during red blood cell intracellular infection

**DOI:** 10.1186/s13071-022-05298-4

**Published:** 2022-05-28

**Authors:** Guang Chen, Shuang-chun Liu, Xiao-yan Fan, Yue-lei Jin, Xin Li, Yun-ting Du

**Affiliations:** 1 Department of Basic Medical Sciences, Taizhou University, No. 1139 Shifu Road, Jiaojiang District, Taizhou, 318000 China; 2 Municipal Hospital Affiliated to Medical School of Taizhou University, No. 381, Zhongshan East Road, Jiaojiang District, Taizhou, 318000 China; 3 Department of Laboratory Medicine, Cancer Hospital of China Medical University–Liaoning Cancer Hospital & Institute, No. 44 Xiaoheyan Road, Dadong District, Shenyang, 110042 China

**Keywords:** *Plasmodium*, Long non-coding RNA, Immune signaling, RBC, Intracellular infection

## Abstract

**Background:**

Parasites interact with their host through “direct” and/or “indirect” mechanisms. *Plasmodium*, for example, either mediates direct physical interactions with host factors or triggers the immune system of the host indirectly, leading to changes in infectious outcomes. Long non-coding RNAs (lncRNAs) participate in regulating biological processes, especially host–pathogen interactions. However, research on the role of host lncRNAs during *Plasmodium* infection is limited.

**Methods:**

A RNA sequencing method (RNA-seq) was used to confirm the differential expression profiles of lncRNAs in *Plasmodium yeolii* 17XL (*P.y*17XL)-infected BALB/c mice. Gene Ontology (GO) and Kyoto Encyclopedia of Genes and Genomes (KEGG) pathway analyses were performed to elucidate the potential functions of *Plasmodium*-induced genes. Subsequently, the effect of specific lncRNAs on the modulation of immune-related signaling pathways in malaria was determined by fluorescence-activated cell sorting, western blot and enzyme-linked immunosorbent assay.

**Results:**

The data showed that in *P.y*17XL-infected BALB/c mice, *Plasmodium* upregulated the expression of 132 lncRNAs and downregulated the expression of 159 lncRNAs. Differentially expressed lncRNAs clearly associated with malaria infection were annotated, including four novel dominant lncRNAs: ENMSUSG00000111521.1, XLOC_038009, XLOC_058629 and XLOC_065676. GO and KEGG pathway analyses demonstrated that these four differentially expressed lncRNAs were associated with co-localized/co-expressed protein-coding genes that were totally enriched in malaria and with the transforming growth factor beta (TGF-β) signaling pathway. Using the models of *P.y*17XL-infected BALB/c mice, data certified that the level of TGF-β production and activation of TGF-β/Smad_2/3_ signaling pathway were obviously changed in malaria infection.

**Conclusions:**

These differentially expressed immune-related genes were deemed to have a role in the process of *Plasmodium* infection in the host via dendritic/T regulatory cells and the TGF-β/Smad_2/3_ signaling pathway. The results of the present study confirmed that *Plasmodium* infection-induced lncRNA expression is a novel mechanism used by *Plasmodium* parasites to modify host immune signaling. These results further enhance current understanding of the interaction between *Plasmodium* and host cells.

**Graphical Abstract:**

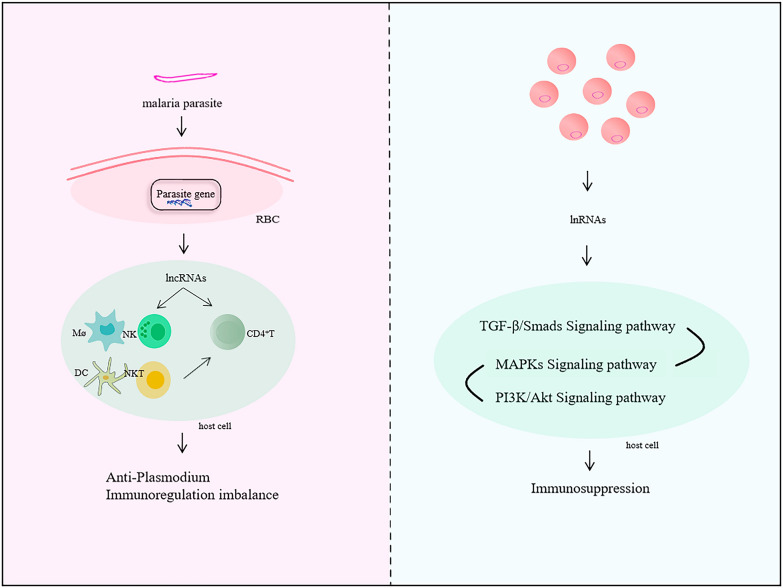

## Background

Malaria remains a serious public health issue around the world, particularly in tropical and subtropical regions. According to the 2020 World Malaria Report of the WHO, there were 229 million people newly infected with malaria in 2019, of whom approximately 409,000 died of severe complications [1]. Of these deaths, 90% occurred in the African continent; in addition, many children and pregnant women experienced life-threatening complications. Despite the use of antimalarial drugs, including artemisinin, the mortality rate among patients with severe malaria is high [2, 3]. Therefore, elucidating the primary factors responsible the development of severe disease, and possibly death, in a subset of infected individuals, while others experience milder infections and recover, is urgently required. An improved understanding of the immune system and other aspects of the physiological balance between the immune system of the host and virulence factors is essential to develop effective interventions to reduce malaria-related morbidity and the fatal consequences of severe complications.

The increasing availability of data on the parasite genome has facilitated the reconstruction of the evolutionary history of the major human malaria parasites and their spread [4]. Long non-coding RNAs (lncRNAs) are RNA fragments > 200 nucleotides in length, and although they do not encode proteins, they can regulate gene expression. The role of lncRNAs in host defense is only starting to be understood in the field of transcriptomics. Thus, current knowledge on the interaction between intracellular pathogens, such as *Plasmodium*, and lncRNAs, is lacking. LncRNAs participate in a wide variety of biological processes and diseases, including cell differentiation, tissue and organ development, flowering in plants and cancer metastasis [5–9]. At present, it is known that lncRNAs participate in transcriptional and post-transcriptional gene regulation. Accumulating research has shown that known lncRNAs usually have a variety of functions, including roles as transcriptional co-activators, recruiters of chromatin modifiers, microRNA sponges and regulators of splicing and mRNA stabilization [10, 11]. While the role of lncRNAs in innate and adaptive immunity is poorly understood, a significant body of evidence suggests that lncRNAs play a significant role in regulating the immunity of the host and its ability to respond against infection [12–14].

In recent years, the host immune response has been found to be associated with the pathogenesis of malaria in the host, which is induced by the upregulation of the type 1 T helper (Th1) cell-mediated inflammatory response. *Plasmodium* infection can also induce protective or pathological responses in the host depending on the immunological system of the host, specifically the level of inflammation (tumor necrosis factor alpha and interferon gamma) and immune response (T cells, T regulatory cells [Tregs] and dendritic cells [DCs]) [15–18]. Numerous types of cells, such as monocytes, macrophages, DCs, neutrophils, T cells and B cells, can express lncRNAs. Xue et al. found that under inflammatory stress, a mass of unannotated long intergenic non-coding RNAs (lincRNAs) were expressed in human blood CD14^+^ monocytes and adipose tissue via de novo RNA sequence assembly [19]. Moreover, interactions between lncRNAs and pathogens regulate these inflammatory-related cells and factors, and then take part in the process of immune responses in the host [20].

Based on these previous findings, the aim of the present study was to confirm the effect of *Plasmodium* on the expression of host lncRNA associated with the immune response. In particular, we study focused on the function of altered lncRNA expression induced by *Plasmodium yeolii* 17XL (*P.y*17XL) infection and the effects on the immune regulation of the infected host. The rodent malaria infection model provides an effective model for understanding the relationship between pathogens and host interaction. Therefore, we infected BALB/c mice with *P.y*17XL and analyzed the altered levels of host lncRNAs following the development of malaria to improve the current understanding of the underlying mechanisms of *Plasmodium* and host interactions in the regulation of anti-malaria immunity.

## Methods

### Construction of the mouse model of parasite infection

Female BALB/c mice aged 6–8 weeks were purchased from the Institute of Zoology (Beijing, China). The animals were kept in the animal care unit under a 12/12-h light–dark cycle at 22–24 °C and 50 ± 5% humidity. The *P.y*17XL strain was provided by Dr. Motomi Torii (Department of Molecular Parasitology, Ehime University Graduate School of Medicine, Ehime, Japan).

Mice were randomly divided into the uninfected (day 0) and *P.y*17XL groups. A mouse model of *Plasmodium* infection was constructed according to a previously described method [21, 22]. BALB/c mice were initially infected via intraperitoneal injection of 1 × 10^6^ *P.y*17XL parasitized erythrocytes. Some mice were used to calculate parasitemia and survival rate. Parasitemia (*n* = 10 mice) was monitored by studying a thin (tail) blood smear stained with Giemsa stain under the light microscope [23]. Mortality (*n* = 10) was monitored daily. Animals were anesthetized with isoflurane (4–5% induction; 2–3% for maintenance, 0.6–0.8 l/min) in a mixture of 0.25% air and 0.5% O_2_; the other mice were used to detect the relative indicators. Spleens were removed, and then all mice were sacrificed by cervical dislocation. All animal experiments were approved by the Animal Experiments Committee of Taizhou University (Approval No. TZXY2019-501).

### Sample collection and preparation

#### RNA isolation, library preparation and sequencing

BALB/c mice infected with *P.y*17XL were sacrified on day 5 post infection (p.i.) and the spleen cells removed. RNA was isolated and the quantity and quality of the isolated RNA determined (Novogene Experimental Department, Novogene, Beijing, China). The NanoPhotometer® spectrophotometer (Implen GmbH. Munich, Germany) was used to confirm RNA purity. The Qubit® RNA Assay Kit with the Qubit® 2.0 Fluorometer (Thermo Fisher Scientific, Waltham, MA, USA) was used to measure RNA concentration. The RNA Nano 6000 Assay Kit with the Bioanalyzer 2100 system (Agilent Technologies, Inc., Santa Clara, CA, USA) was used to determine RNA integrity.

#### Library preparation for lncRNA sequencing (Novogene)

A total of 20 ng RNA per sample was used for the preparation of the RNA samples. As a first step, ribosomal RNA (rRNA) was removed using the Epicentre Ribo-Zero™ RRNA Removal Kit (Illumina, Inc., San Diego, CA, USA), following which the free rRNA residue was removed using the ethanol precipitation technique. Next, the Illumina® NEBNext® Ultra™ II Directional RNA Library Prep Kit (New England BioLabs, Inc., Ipswich, MA, USA) was used to generate sequencing libraries from rRNA-depleted RNA according to the manufacturer’s protocols. Briefly, the rRNA-depleted RNA was fragmented using divalent cations at high temperatures in NEBNext First Strand Synthesis Reaction Buffer (5×). First, chain complementary DNA (cDNA) was synthesized using random hexamer primers and m-mulv reverse transcriptase (RNaseH-). Secondly, strand cDNA was synthesized using DNA polymerase I and RNase H. In the reaction buffer, dUTP replaced dNTPs and dTTP. The remaining suspension was converted to a blunt end by exonuclease/polymerase activity. After the 3′-end of the DNA fragment was adenylated, the NEBNext adaptor with hairpin ring structure was linked in preparation for hybridization.

To optimize cDNA fragments to lengths ranging from 150 to 200 bp, the library fragments were purified using AMPure XP (Beckman Coulter, Inc., Brea, CA, USA).The size was then selected using 3 μl User Enzyme (New England BioLabs, Inc.), and the adapter ligated cDNA was incubated at 37 °C for 15 min, then at 95 °C for 5 min, followed by PCR.

Phusion™ high-fidelity DNA polymerase, universal PCR primers and index (X) primers were used for the PCR. Finally, the product was purified using the AMPure XP system and library quality was evaluated on the Agilent 2100 system (Agilent Technologies, Inc.).

#### Clustering and sequencing (Novogene)

On a cBot Cluster Generation System, the index-coded samples were clustered using the TruSeq PE Cluster Kit v3-cBot-HS (Illumina, Inc.) according to the manufacturer’s instructions. After cluster generation, the libraries were sequenced on an Illumina HiSeq 2500 platform and 125-bp paired-end reads were generated.

### Data analysis (Novogene)

#### Quality control

Raw data in FastQ format (raw read) were first processed through an internal Perl script. In this step, clean data (clean reads) were obtained from raw data by removing reads containing adapters, ploy-N and low-quality reads. The clean data were then used to calculate the Q20 and Q30 content, and the clean data with high quality was the basis of all the downstream analyses.

#### Mapping to the reference genome

Based on the genome website, the reference genome and the gene model annotation files were downloaded directly. HISAT2 v2.0.4 was used to build an index of the reference genome and to compare end clean reads with the reference genome [24]. HISAT2 was run with ‘–rna-strandness RF’; other parameters were set as the default.

#### Transcriptome assembly

The mapped reads of each sample were assembled using String Tie (v1.3.3) [25] in a reference-based approach. String Tie uses a novel network flow algorithm and an optional de novo assembly step to assemble and quantify full-length transcripts representing multiple splice variants for each gene locus.

#### Coding potential analysis

##### Coding-Non-Coding-Index

Coding-Non-Coding-Index (CNCI) (v2) profiles to adjoin nucleotide triplets were constructed to effectively distinguish protein-coding and non-coding sequences independent of known annotations [26]. The CNCI was used with default parameters.

##### Coding potential calculator algorithm version 2

Using the coding potential calculator algorithm version 2 (CPC2) (v0.1), we adopted four intrinsic features of the sequence that are easy to understand and have biological significance. At the DNA level, the Fecht fraction was used to capture the vantage point of each base in the sequence. At the RNA level, the length and integrity of the open reading frame (ORF) is strong, and those correlated with protein-encoded transcripts are more likely to have a long, high-quality ORF.

Moreover, based on the assumption that the hypothetical peptide identified in a non-coding transcript should have different chemical properties than the real ones encoded by real coding sequences, CPC2 also added several peptide level features into the candidate list and eventually adopted the isoelectric point (pI) in the final support vector machine (SVM) model [27]. Using Pfam-scan (v1.3), we translated each transcript from all three possible frameworks and identified the presence of any known protein family domain recorded in the Pfam database (release 27; Pfam A and Pfam B) [28]. Any transcript with a Pfam hit was excluded from the following steps. Pfam searches use default parameters of −E 0.001 to −domE 0.001[29].

##### Phylogenetic codon substitution frequency

Evolutionary signatures characteristic to alignments of conserved coding regions were examined using phylogenetic codon substitution frequency (PhyloCSF) (v20121028), including the high frequencies of synonymous codon substitutions and conservative amino acid substitutions, and the low frequencies of other missense and non-sense substitutions, to distinguish protein-coding and non-coding transcripts [30]. We built multi-species genome sequence alignments and ran phyloCSF with default parameters.

Any or all of the transcripts predicted to have coding potential from the aforementioned four tools were filtered out, and those transcripts without coding potential were our candidate lncRNA set.

#### Target gene prediction

The cis role of the target gene was predicted. Cis-action is the action of lncRNA on adjacent target genes. We searched the upstream and downstream 10 k/100 k encoding genes of lncRNA and then analyzed their functions.

The trans role of the target gene was also predicted. Trans roles are recognized by lncRNAs through their expression levels. We calculated the expressed correlation between lncRNA and the R functional coding gene "cor.test".

#### Quantification of transcript expression level

StringTie (v2.1.1) was used to calculate fragments per kilobase of transcription per million mapped reads (FPKMs) of both lncRNAs and coding transcripts in each sample [25]. FPKM refers to fragments per kilobase exon per million fragments, mapped to the fragment according to the length of the fragment and read count.

#### Differential expression analysis

The Ballgown suite has numerous types of functions, including interactive exploration of the transcriptome assembly, visualization of the transcriptome structure and per-site eigen-specific abundance and the assembly feature versus the annotation feature’s post-annotation function [31]. Transcripts with an adjusted *P*-value < 0.05 or were assigned as differentially expressed.

The Cuffdiff tool provides statistical routines for determining differential expression in digital transcript or transcript expression data using a model based on the negative binomial distribution [32]. Transcripts with adjusted *P*-value < 0.05 or *P*-value < 0.05 were assigned as differentially expressed.

EdgeR is a Bioconductor software package designed to detect differential expression of replicated count data. A Poisson model of overdispersion is used to explain biological and technical variability. Empirical Bayes methods are used to moderate the degree of overdispersion across transcripts, thus improving the reliability of inference. As long as at least one phenotype or experimental condition is replicated, the method can be used with even a minimal level of replication [33]. Transcripts with an adjusted *P*-value < 0.05 or *P*-value < 0.05 were assigned as differentially expressed.

#### Gene Ontology and Kyoto Encyclopedia of Genes and Genomes enrichment analysis

The GoSeq R package was used for Gene Ontology (GO) enrichment analysis of differentially expressed genes or lncRNA target genes to correct for gene length bias [34]. GO genes with a corrected *P*-value < 0.05 were considered to be significantly enriched in differentially expressed genes. Kyoto Encyclopedia of Genes and Genomes (KEGG) is a database resource for understanding advanced functionality and utility of biological systems [35], such as cells, organisms and ecosystems, assembled at the molecular level, especially from large-scale molecular datasets produced by genome sequencing and other high-throughput experimental techniques (http://www.genome.jp/kegg/). KOBAS software was used to detect the statistical enrichment of differentially expressed genes or lncRNA target genes in the KEGG pathway [35, 36].

### Cell-surface staining, intracytoplasmic staining and flow cytometry

BALB/c mice were sacrificed at the indicated time to analyze dynamic changes in splenic DCs (myeloid [mDCs] and plasmacytoid [pDCs]), specifically in the population of major histocompatibility complex class II (MHCII) and CD80 molecules on CD11c^+^DCs, the populations of Tregs. Unless otherwise indicated, antibodies were purchased from BD Biosciences (Franklin Lakes, NJ, USA).

Spleen cells from BALB/c mice were prepared at different time points after infections. To assess DCs, 1 × 10^6^ cells were stained with FITC-conjugated CD11c monoclonal antibody (mAb) (clone HL-3) and PE-conjugated anti-CD11b (clone M1/70), PerCP-conjugated-CD45R/B220 (clone RA3-6B2), APC-conjugated anti-MHCII (clone M5/114.15.2; eBioscience, Thermo Fisher Scientific, Waltham, MA, USA) and PerCP-conjugated anti-CD80 (clone 16-10A1) [37].

To assess Tregs, FITC-conjugated anti-CD4 (clone GK1.5) and PE-conjugated anti-CD25 antibodies (clone PC61) were added to 1 × 10^6^ spleen cells, which were resuspended in 100 μl of phosphate buffered saline (PBS) supplemented with 1% FCS for surface staining. The cells were then fixed and permeabilized, and intracytoplasmic staining was performed using APC-conjugated anti-Foxp3 (clone FJK16s; eBioscience) antibody [37]. The cells were then washed twice with PBS containing 1% FCS and suspended in 300 μl of PBS. The cells were analyzed in a FACSCalibur cytofluorometer using CellQuest software (BD Biosciences).

### Ezyme-linked immunosorbent assay

For the quantification of cytokines, splenocytes were harvested from mice at the indicated time points (day 0, 3 and 5) [37]. Spleen cells were adjusted to a final concentration of 10^7^ cells/ml in RPMI-1640 supplemented with 10% heat-inactivated FCS. Aliquots (500 µl/well) of the cell suspensions were incubated in 24-well flat-bottom tissue culture plates (Falcon®) in triplicate for 48 h at 37 °C in a humidified 5% CO2 incubator. Supernatant fractions were collected and stored at − 80 °C for use in the assays for detecting cytokines.

Transforming growth factor beta (TGF-β) expression was measured using commercial enzyme-linked immunosorbent assay (ELISA) kits, according to the manufacturer's protocol (R&D Systems, Inc. Minneapolis, MN, USA). The OD values were measured using a microplate reader at 450 nm. The cytokine concentration of each sample was calculated using a standard curve generated using recombinant cytokines.

### Western blot analysis

Protein was extracted from the spleen of BALB/c mice infected with *P.y*17XL. Protein samples were resolved by 10% sodium dodecyl sulfate-polyacrylamide gel electrophoresis and transferred to PVDF membranes (EMD Millipore, MilliporeSigma, Burlington, MA, USA). Membranes were blocked in 5% nonfat dry milk and subsequently incubated with primary antibodies against p-Smad2/3/Smad2 (1:1000; HUABIO, Woburn, MA, USA), TGF-β (1:1000; Abcam, Oxford, UK) and β-actin (1:1000; CST Biological Reagents Co., Ltd., Shanghai, China) overnight. Horseradish peroxidase-conjugated anti-rabbit IgG (1:2000; CST Biological Reagents Co., Ltd.) were used as the secondary antibodies. PVDF membranes were developed using the Image-Pro Plus system (Media Cybernetics, Inc., Rockville, MD, USA).

## Results

### *P.y*17XL infection course in BALB/c mice

**Fig. 1:**
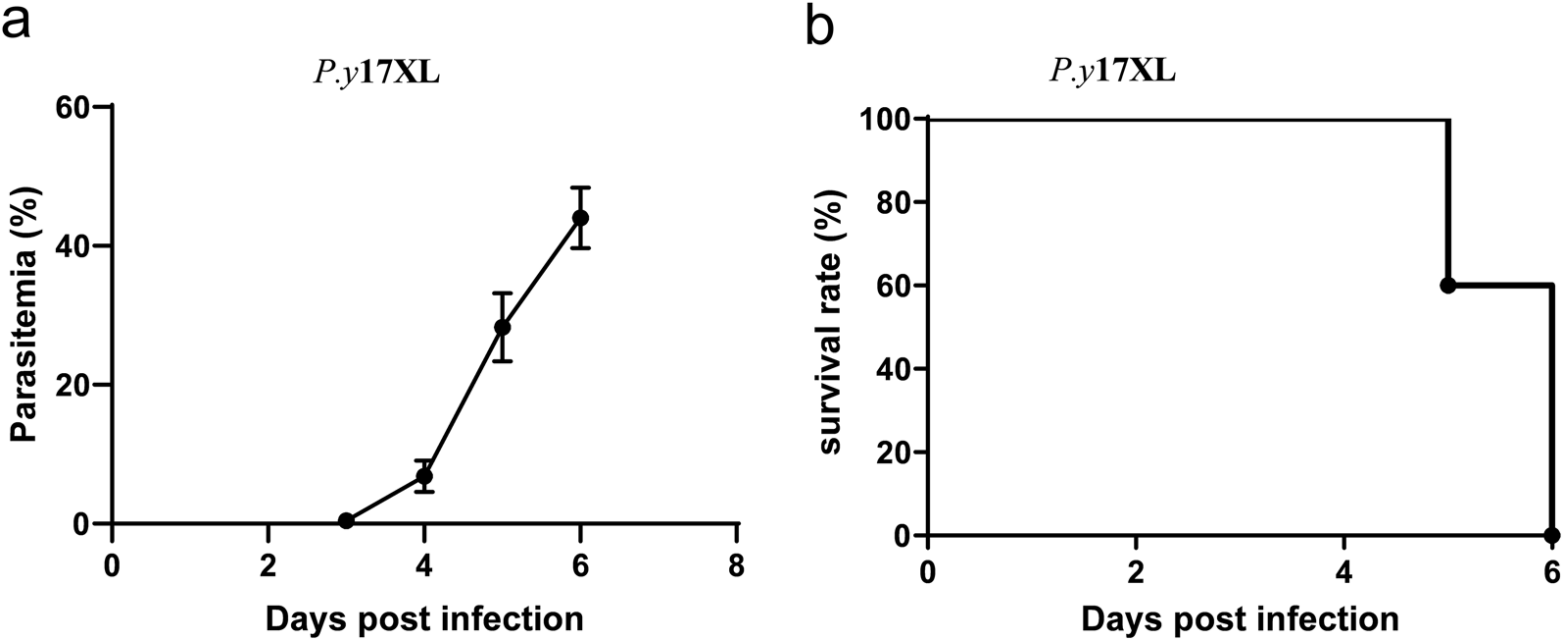
Parasitemia in (**a**) and survival rate of (**b**) *P.y*17XL-infected BALB/c mice. Parasitemia was calculated by counting the number of parasite-infected erythrocytes per 1000 erythrocytes. Mortality was monitored daily. Data represent three separate experiments. Abbreviations: *P.y*17XL, *Plasmodium yeolii* 17XL

It is well known that different rodent models develop different outcomes when infected with the same strain of malaria parasite. In this study, BALB/c mice were infected with *P.y*17XL. As expected, *P.y*17XL-infected BALB/c mice developed a high parasitemia on day 6 p.i. with a peak around 39–49%, and all mice died (Fig. 1a, b).

### Differential expression analysis of lncRNAs in *Plasmodium-*infected mice

**Fig. 2:**
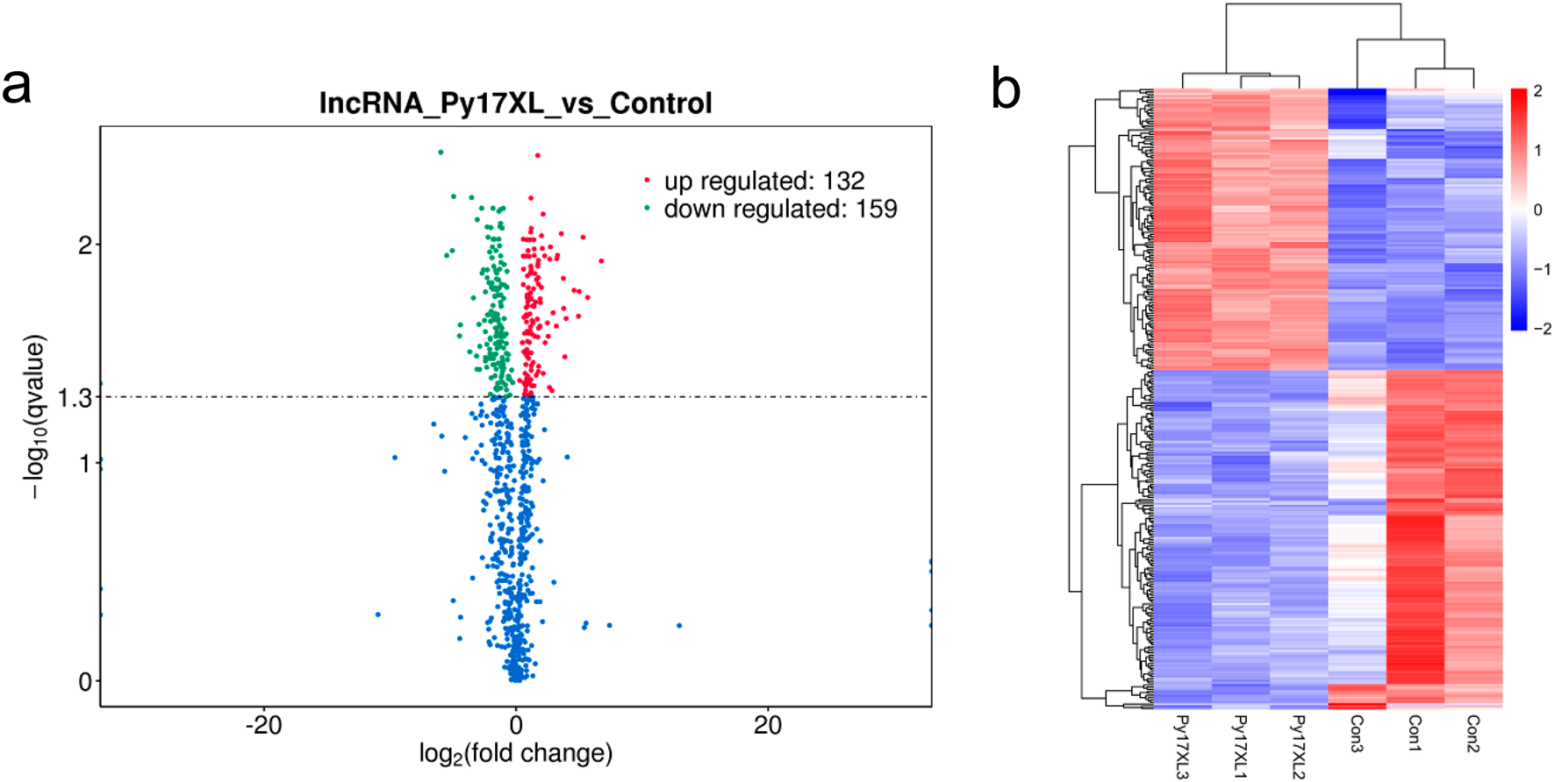
Differential expression analysis of lncRNAs in *Plasmodium*-infected mice. **a** Volcano plot filtering to visualize fold regulation and statistical significance in lncRNAs. Statistically significant (*P* < 0.05) upregulated (red) or downregulated (green) expression changes are shown in BALB/c mice infected with *P.y*17XL compared with normal control. **b** Hierarchical clustering of differentially expressed lncRNAs for uninfected vs. *P.y*17XL infection. Values in the color scale are normalized intensities. Red bands indicate high relative expression, and blue bands indicate low relative expression. Abbreviations: Con, Control; lncRNA, long non-coding RNA; TUCP, transcripts of uncertain coding potential

The differential expression of lncRNAs were analyzed using the volcano map (Fig. 2a). The default screening threshold was set as* q*-value < 0.05 (if too few differential genes were screened using* q*-value < 0.05, *P* < 0.05 was adopted for screening differential genes). Compared with the normal group, *P.y*17XL infection upregulated the expression of 132 lncRNAs and downregulated the expression of 159 lncRNAs.

The expression patterns of different transcripts were determined using clustering analysis in different experimental groups. FPKM values of varying transcripts in different experimental groups were considered to be expression levels, and hierarchical clustering analysis was performed. Different colors were used to represent the grouping information from different experimental groups, and similar expression patterns within the same group may have similar functions or participate in the same biological processes (Fig. 2b). In this study, we found that *P.y*17XL infection reversed the expression of the different transcripts compared with expression in normal (control) mice, thereby demonstrating the obviously differential expression of lncRNAs (Table 1).

**Table 1:** Differentially expression of long non-coding RNAs

LncRNA ID	LncRNA gene ID	LncRNA gene symbol	LncRNA status	*P.y*17XL	Control	Log2 fold change	*P*-value	*q*-value
ENSMUST00000213884.1	ENSMUSG00000111521.1	RP23-228B2.5	Annotated_lncRNA	4.073482	2.644538667	0.623246464	0.01290096	0.043118944
LNC_003011	XLOC_038009		Novel_lncRNA	9.578093667	25.069341	− 1.388113623	0.001486492	0.014573034
LNC_004637	XLOC_058629		Novel_lncRNA	3.792462333	1.414468333	1.422874973	0.013605837	0.044492281
LNC_005184	XLOC_065676		Novel_lncRNA	0.901344	4.008685333	− 2.152979452	0.001599882	0.015011419

*LncRNA* Long non-coding RNA, *P17XL .yPlasmodium yeolii* 17XL

### LncRNA-targeted mRNA prediction

LncRNAs have no coding potential, and they function by regulating related genes. At present, the underlying mechanisms of lncRNA and mRNA interactions are unclear, and the biological function of lncRNA is predicted by its co-localization and co-expression with protein-coding genes. It has been reported that lncRNAs may have a regulatory effect on adjacent protein-coding genes. We set the threshold value of co-localization as 100 kb upstream and downstream of the lncRNA [38]. We then predicted the main function of the differential lncRNA associated with *Plasmodium* infection by functional enrichment analysis of the mRNA genes of lncRNA co-localization and lncRNA co-expression (Table 2).

**Table 2:** Prediction of four differentially expressed lncRNA-targeted mRNAs

LncRNA ID	LncRNA gene ID	LncRNA gene symbol	LncRNA status	mRNA ID	mRNA gene ID	mRNA gene symbol	Distance (kb)	Location
ENSMUST00000213884.1	ENSMUSG00000111521.1	RP23-228B2.5	Annotated_lncRNA	ENSMUST00000003687	ENSMUSG00000021253	Tgfb3	3540	Upstream
LNC_003011	XLOC_038009		Novel_lncRNA	ENSMUST00000167924	ENSMUSG00000024401	Tnf	32,320	Downstream
LNC_004637	XLOC_058629		Novel_lncRNA	ENSMUST00000003687	ENSMUSG00000021253	Tgfb3	3220	Upstream
LNC_005184	XLOC_065676		Novel_lncRNA	ENSMUST00000003687	ENSMUSG00000021253	Tgfb3	3094	Upstream

### GO and KEGG enrichment analysis of differentially expressed lncRNA target genes

**Fig. 3:**
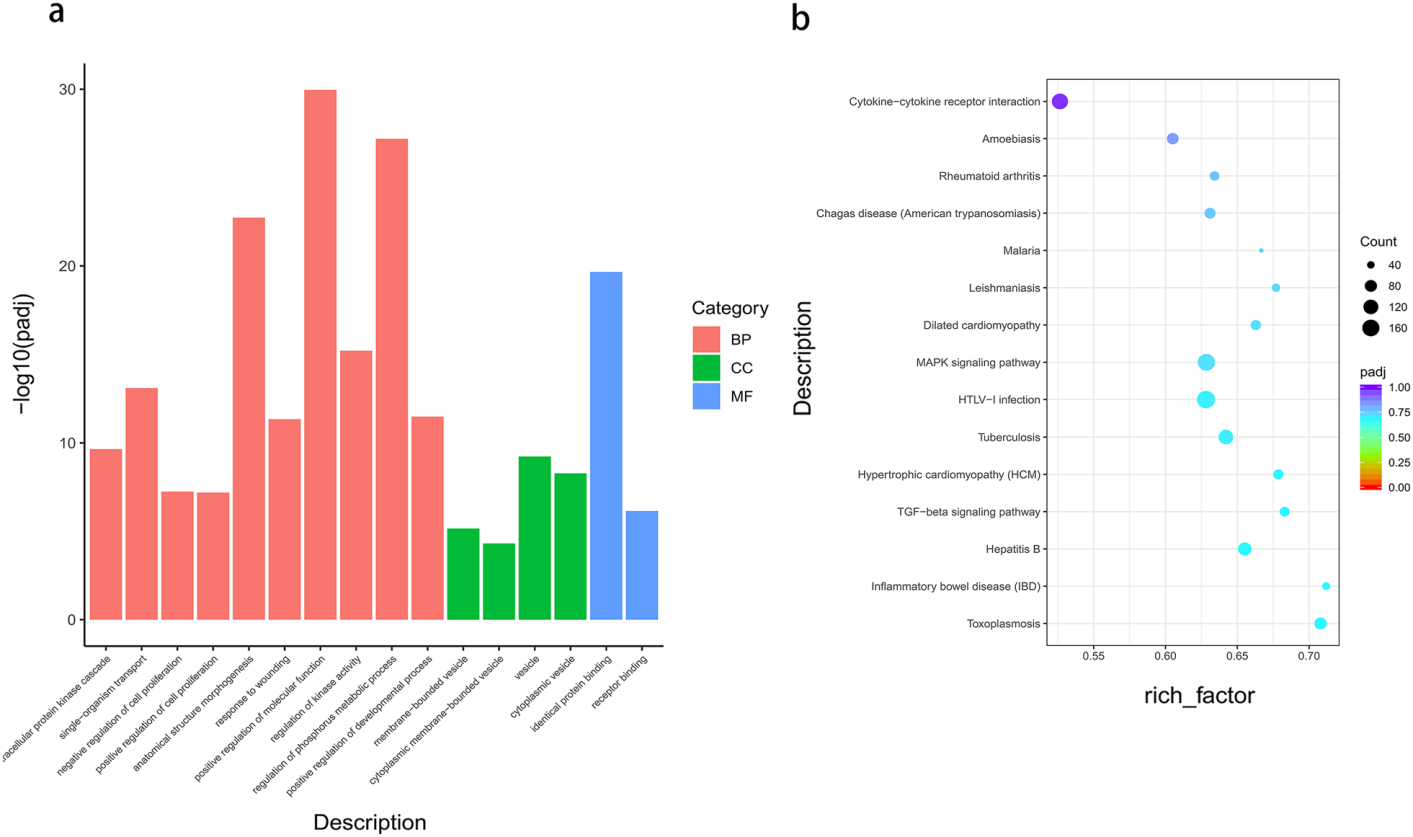
GO enrichment and KEGG pathway analyses of target genes of four differentially expressed lncRNAs in *P.y*17XL infected mice. **a** The enriched GO terms in the biological process, cellular component and molecular function categories of target genes of four differentially expressed lncRNAs. **b** Scatterplot of KEGG pathway analysis of the predominant pathways. The* X*-axis refers to pathway enrichment; the* Y*-axis represents the names of the significantly enriched pathways. The *P*-values are expressed by variations from red to yellow. A deeper yellow color indicates a greater significant difference. Abbreviations: GO, Gene Ontology; KEGG, Kyoto Encyclopedia of Genes and Genome

We used GO analysis to assess the enrichment of four differentially expressed lncRNAs found in the RNA sequencing (RNA-seq) results in the categories biological process, cellular component and molecular function. Among these, four differentially expressed lncRNAs were significantly enriched in biological process and cellular component, such as positive regulation of molecular function and regulation of phosphorus metabolic process and vesicle (Fig. 3a).

We used KEGG pathway enrichment analysis to further determine the potential biological functions of the aforementioned differentially expressed lncRNAs, and then their pathways and molecular interactions were predicted. Figure 3b shows the most enriched pathways, which involved included malaria infection, the TGF-β signaling pathway and cytokine-cytokine receptor interaction (Fig. 3b).

### LncRNA target-immunity relative network analysis

**Fig. 4:**
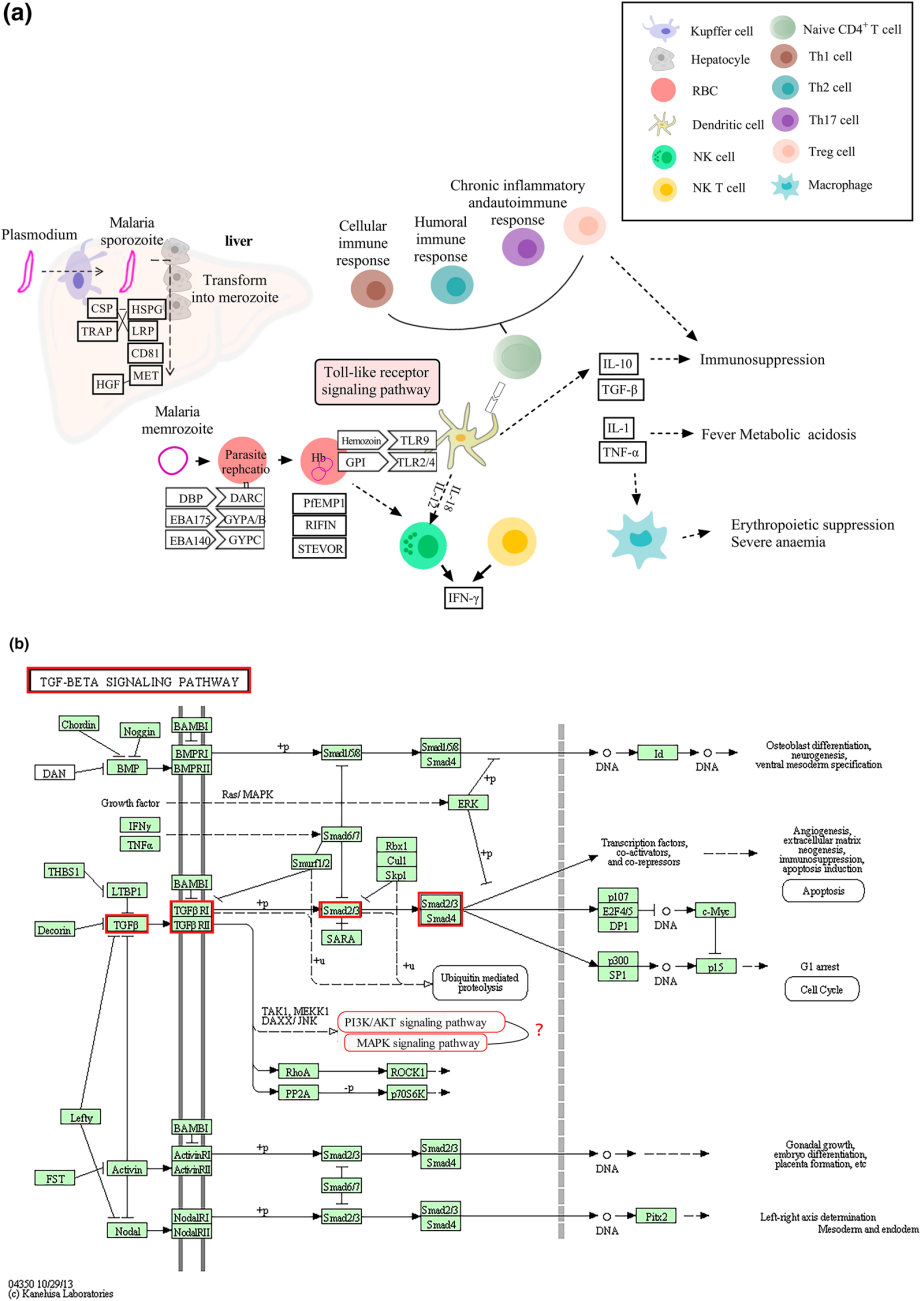
Target-immunity relative network analysis of specific lncRNAs in BALB/c mice infected with *P.y*17XL. **a** Signaling pathway associated with malaria, **b** TGF-β signaling pathway

KEGG analysis revealed four differentially expressed lncRNAs that co-localized/were expressed with protein-coding genes that were totally enriched in malaria infection and the TGF-β signaling pathway. To further clarify the regulatory functions of these differentially expressed lncRNAs in malaria, we determined the distribution of differentially expressed genes in the pathway map (Fig. 4a and b). These results hinted at these four differentially expressed lncRNAs targeting genes participating in the occurrence and development of malaria infection through regulation of the TGF-β signaling pathway.

### Analysis of the characteristics of immunity in*P.y*17XL-infected BALB/c mice

**Fig. 5:**
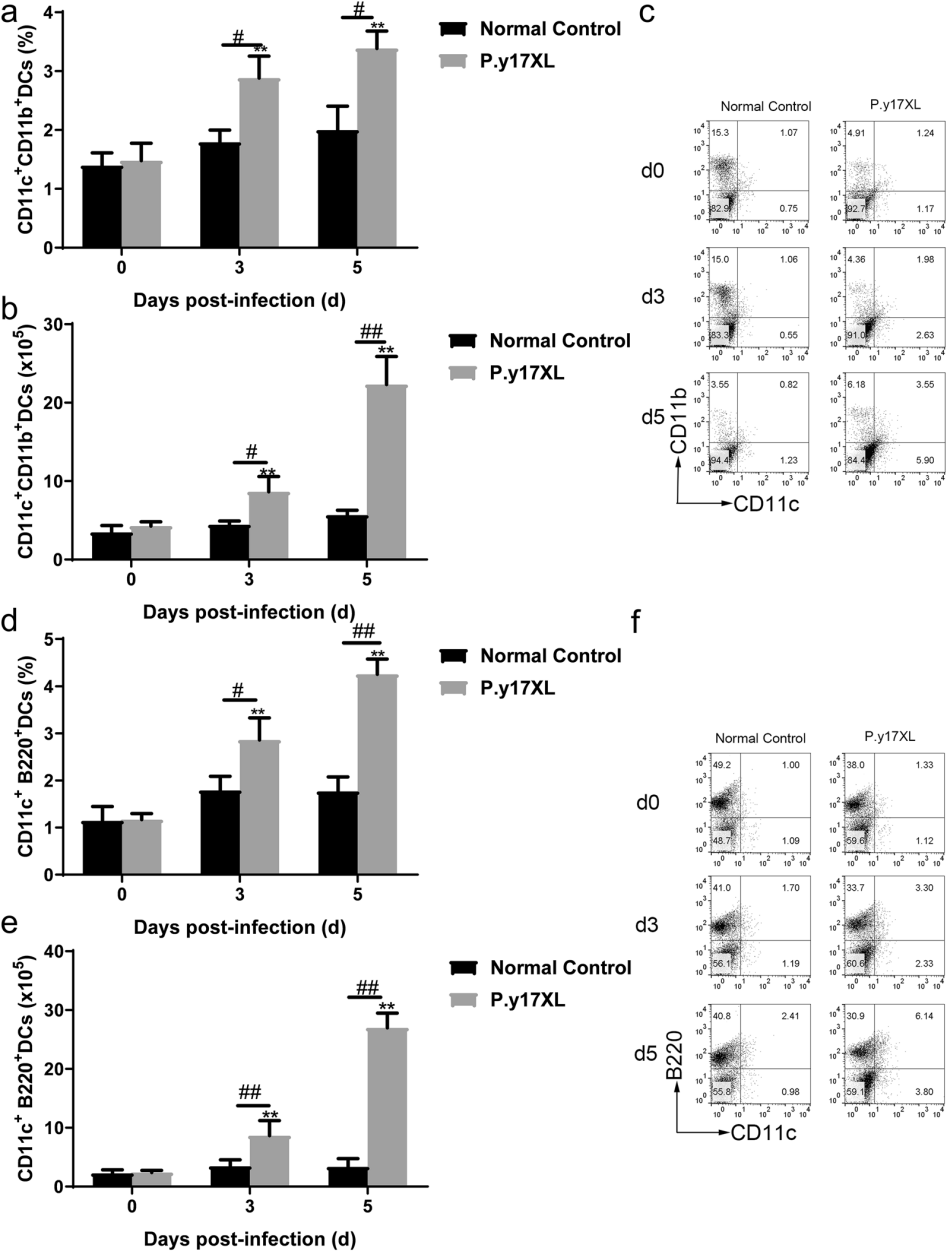
Characteristics of the subsets of DCs during *P.y*17XL infection. Double staining with FITC-anti-CD11c, PE-anti-CD11b and PerCP-anti-CD45RO/B220 was used to detect the proportion, absolute cell numbers (column diagram, left) and representative dot plots (right) of mDCs (**a**–**c**) and pDCs (**d**–**f**) via flow cytometry in BALB/c mice. Results are shown as the mean of five mice per group ± SEM. Asterisks indicate significant difference at *P < 0.05 and **P < 0.01 vs. control mice (non-infected mice, 0 days); hashtags indicate significant difference at ^#^*P* < 0.05 and ^##^*P* < 0.01 in mice at 3 and 5 days p.i. Abbreviations: d, Days; DCs, dendritic cells; mDCs, myeloid DCs; pDCs, plasmacytoid DCs, p.i., post inoculation; SEM, standard error of the mean

**Fig. 6:**
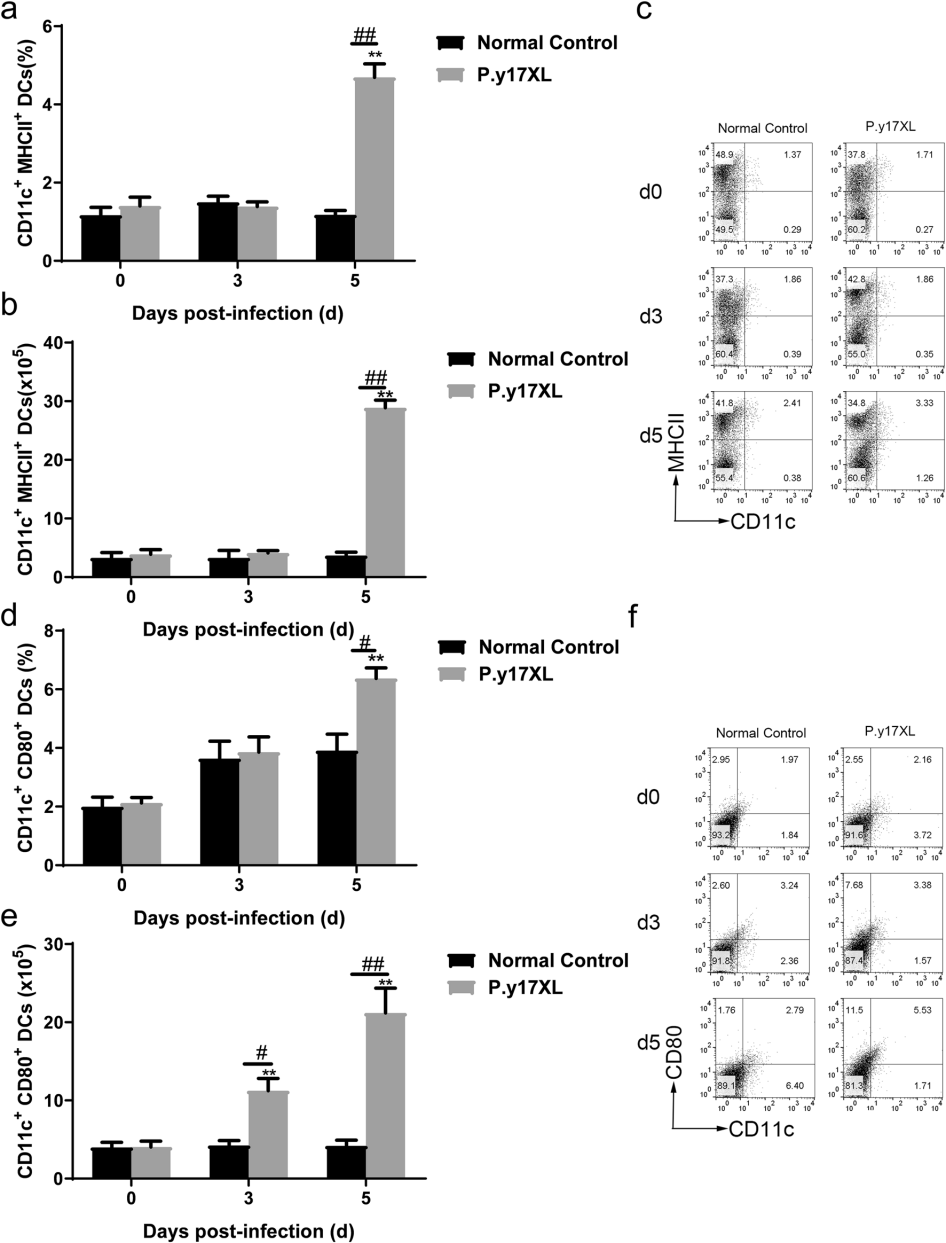
Characteristics of the surface molecular expression of DCs during *P.y*17XL infection. Double staining with FITC-anti-CD11c, APC-anti-MHCII and PerCP-anti-CD80 was used to detect the proportion, absolute cell numbers (column diagram, left) and representative dot plots (right) of MHC II (**a–c**) and CD80 (**d**–**f**) on CD11c^+^DCs via flow cytometry in BALB/c mice. Results are shown as the mean of five mice per group ± SEM. Asterisks indicate significant difference at *P < 0.05 and **P < 0.01 vs. control mice (non-infected mice, 0 days); hashtags indicate significant difference at ^#^*P* < 0.05 and ^##^*P* < 0.01 in mice at 3 vs. 5 days p.i. Abbreviations: MHCII, Major histocompatibility complex II

**Fig. 7:**
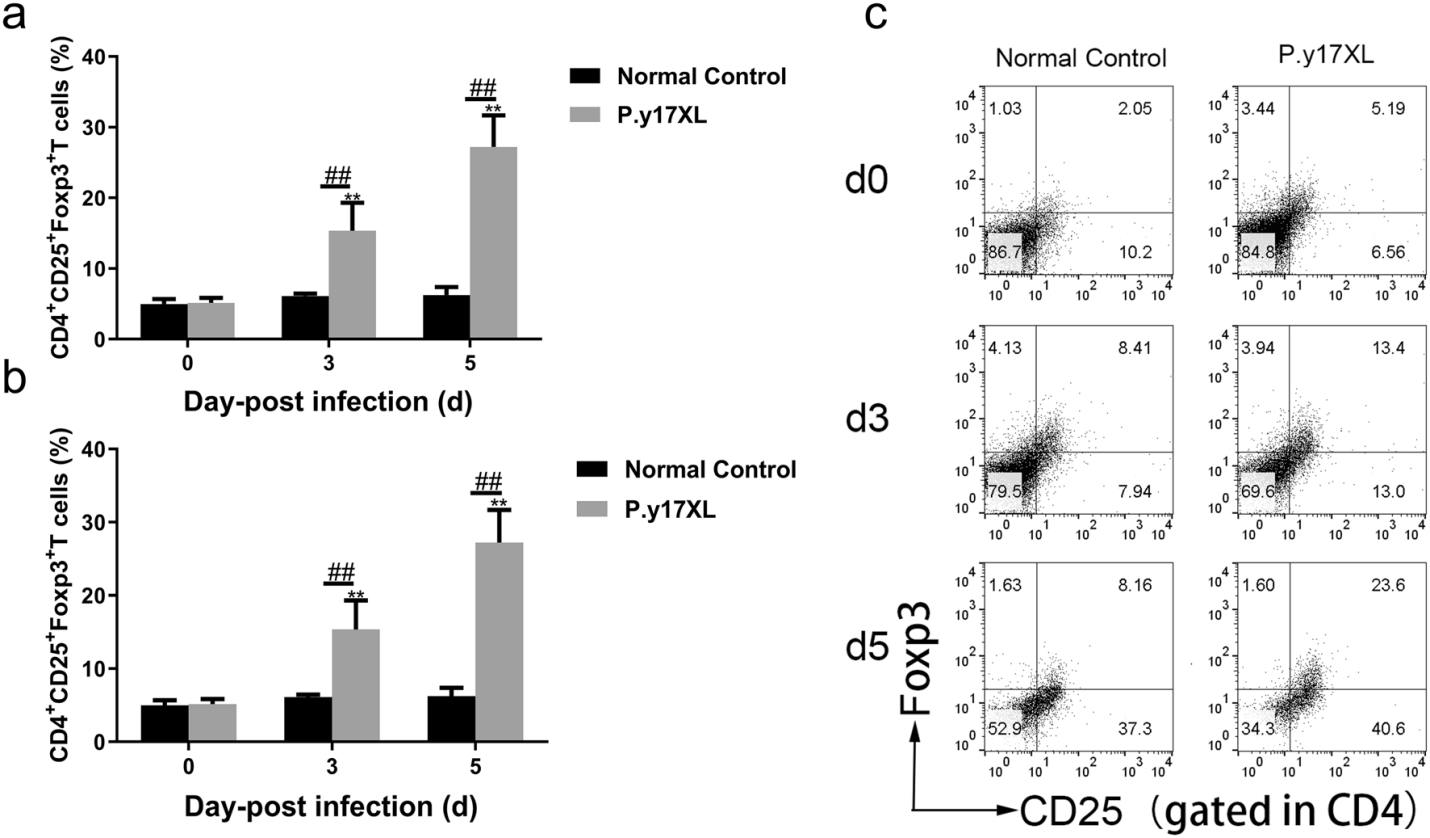
Characteristics of the subsets of Tregs during *P.y*17XL infection. Triple staining with FITC-anti-CD4, PE-anti-CD25 and APC-anti-Foxp3 was used to detect the proportion (**a**), absolute cell numbers (**b**) (column diagram, left) and representative contour (right) of Tregs (**c**) in *P.y*17XL-infected BALB/c mice. Results are shown as the mean of four mice per group ± SEM. Asterisks indicate significant difference at *P < 0.05 and **P < 0.01 vs. control mice (non-infected mice, 0 days); hashtags indicate significant difference at ^#^*P* < 0.05 and ^##^*P* < 0.01 in mice at 3 vs. 5 days p.i, using one-way analysis of variance. Abbreviations: Tregs, T regulatory cells

Based on the results of our KEGG pathway analysis, regulation of DCs and Tregs by specific lncRNAs occurs during the infectious process of malaria in *P.y*17XL-infected BALB/c mice. In the present study, we detected the number of DCs and Tregs, and explored their potential functions in order to clarify the characteristics of immunity in *P.y*17XL-infected BALB/c mice. Compared with the normal (control) group, it was notable that *P.y*17XL infection consistently increased the percentages and absolute cell numbers of mDCs (Fig. 5a–c) and pDCs (Fig. 5d–f) on days 3 and 5 p.i. (*P* < 0.05). At the same time, we also found that *P.y*17XL infection markedly increased the expression levels of MHCII and CD80 molecules (*P* < 0.05; Fig. 6a–f) on DCs on day 5 p.i., compared with the normal (control) group. Consistently, *P.y*17XL infection also increased the percentages and absolute cell numbers of Tregs (Fig. 7a–c) on days 3 and 5 p.i., compared with the normal group (*P* < 0.01). These results demonstrated that *Plasmodium* infection clearly altered the immune response pattern of the host, which was associated with the interaction between *Plasmodium* and the host.

### Analysis of the characteristic signaling pathways in *P.y*17XL-infected BALB/c mice

**Fig. 8:**
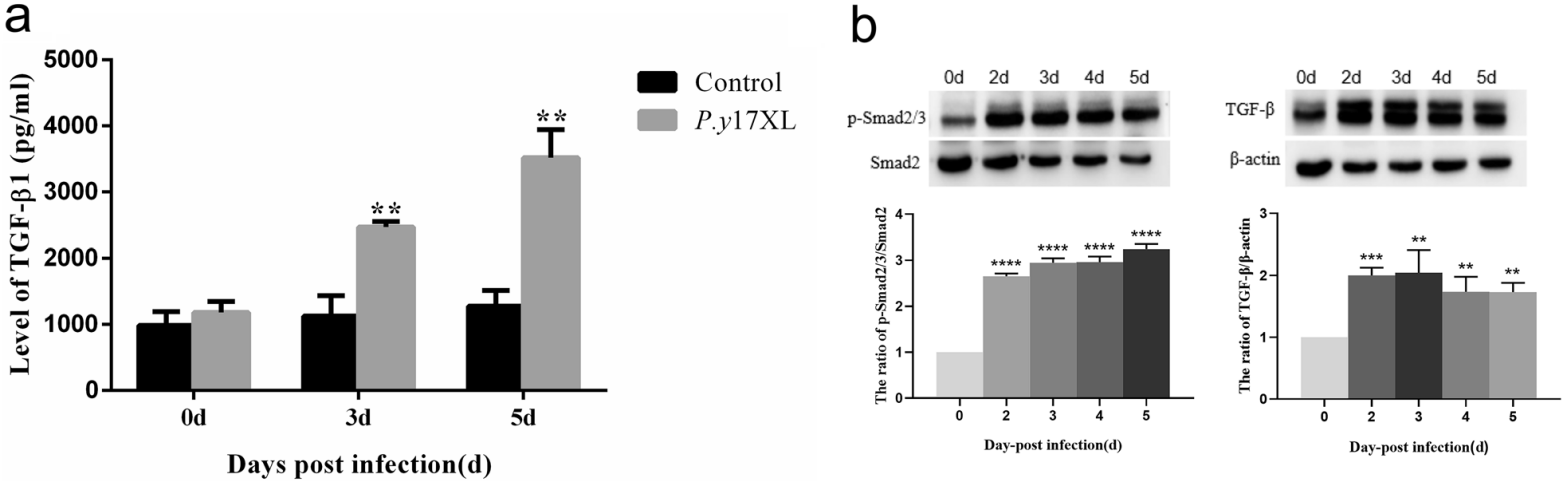
Characteristics of the TGF-β signaling pathway in BALB/c mice infected with *P.y*17XL. **a** ELISA was performed to detect the levels of TGF-β in the supernatants of cultured spleen cells in BALB/c mice infected with *P.y*17XL. **b** TGF-β1,Smad2/3 and p-Smad2/3 protein expression levels in BALB/c mice infected with *P.y*17XL. The results of three independent repeats are shown. Data are presented as the mean ± SD. Asterisks indicate significant difference at *P < 0.05 and **P < 0.01 vs. baseline levels. Abbreviations: ELISA, Enzyme-linked immunosorbent assay; p-, phosphorylated; TGF-β, transforming growth factor beta

Based on the results of the KEGG pathway analysis, TGF-β and the related signaling pathway regulated by specific lncRNAs are involved in the infectious process of malaria in *P.y*17XL-infected BALB/c mice (Fig. 4b). We thus designed an experiment to detect the level of TGF-β1 production in the supernatant samples using ELISA in *P.y*17XL-infected BALB/c mice. The level of TGF-β1 production increased on days 3 and 5 p.i. (*P* < 0.01), increasing to levels that were threefold higher than in normal mice (Fig. 8a). We then analyzed the activity of the TGF-β/Smad2/3 signaling pathway in *P.y*17XL-infected BALB/c mice and found that the expression levels of TGF-β and p-Smad2/3 protein were notably increased on days 2–5 p.i (Fig. 8b). These results indicate that *Plasmodium* infection activated the TGF-β/Smad2/3 signaling pathway and participates in the regulation of anti-malaria immunity.

## Discussion

Parasites, which can induce serious diseases and high mortality rates, remain a serious threat to human health worldwide [39]. Following infection, these pathogens can deplete the nutrients of the host, while at the same time also dysregulate the metabolic processes and immune system of the host [40]. *Plasmodium* is one of the oldest recorded parasites infecting humans and has proved particularly difficult to eradicate. The success of this parasite is a result of its ability to evade the human immune system and use the responses of the host as a physiological signal to regulate its life-cycle, allowing the parasite to quickly adapt to its immediate host environment [41]. Controlling the environment of the host is a crucial step for *Plasmodium,* which is a model of invasion and survival in host cells. It is increasingly recognized that the interaction between parasite proteins and host factors is necessary to establish infection and virulence at every stage of the parasite life-cycle [41].

LncRNAs are involved in interactions between multiple species, such as, for example, vector-host–pathogen interactions. It is worth nothing that lncRNAs can be either vector/host-derived or encoded by pathogens [42]. In the pathogen and host interactions, there are numerous types of mechanisms, including regulation of pathogen growth and replication or cellular homegrown defense. Recent research has shown that the characterization of lncRNAs and their targets in different species is the key factor to understanding the function of these non-coding RNAs in interspecies crosstalk. LncRNAs exist in the nucleus and cytoplasm and can function as scaffolds or decoys [43]. LncRNA is a key regulator of gene expression and is involved in most cell processes [42]. In the present study, we comprehensively studied the host lncRNA regulated by *P.y*17XL during intracellular infection using a RNA-seq method. We identified approximately 132 potentially upregulated lncRNAs and approximately 159 downregulated lncRNAs associated with *Plasmodium* infection. In fact, we have already identified an annotated and three novel dominant lncRNAs associated with immunological pathogenesis of *Plasmodium* infection, including ENMSUSG00000111521.1, XLOC_038009, XLOC_058629 and XLOC_065676; these provide a rich basis for further analysis (Table 1). Although some of the lncRNAs we identified may be induced by other factors, they still have the potential to be key factors during *Plasmodium* infection-induced immunity.

LncRNAs are attracting increased attention as emerging mediators that extensively regulate cellular signaling and gene expression. In our study, we detected a subset of parasite-regulated lncRNAs that participate in encoding immune-related genes. LncRNAs have a key regulatory role during parasite invasion of the host. In other words, lncRNAs regulate gene transcription, mRNA stability and translation and the immune response in parasites and hosts [44]. Based on our results (Table 1), we predicated potential targeted mRNAs of these differentially lncRNAs in order to analyze the biological function of lncRNAs by co-localization/co-expression with protein-coding genes (Table 2). GO and KEGG enrichment analysis revealed that four differentially lncRNAs participated in the regulation of the expression of transcripts associated with the host immune response, cell proliferation, metabolism and diseases. Specifically, the differentially expression of lncRNAs induced by *Plasmodium* infection modified the characteristic of immune respones via the TGF-β signaling pathway in malaria infection (Fig. 3). These results suggest that these lncRNAs were closely related to the immune response of the host. Thus, we obtained novel insights into the interactions between *Plasmodium* and host mediated by lncRNAs.

In the present study, we found that the four differentially lncRNAs target genes were simultaneously enriched in the “malaria and TGF-β signaling pathway” associated with the immune response in the course of *Plasmodium* infection (Fig. 4). We then sought to determine whether these specific pathways take part in immune regulation in *P.y*17XL-infected BALB/c mice. Combined with KEGG analysis, we found that *P.y*17XL infection altered the characteristics of circulating DCs and Tregs in *P.y*17XL-infected BALB/c mice (Figs. 5–7), which also involved the differentially expressed lncRNA. DCs, which are considered to be professional antigen presenting cells, are highly specialized in presenting antigens to T cells [45], and then in activating CD4^+^ T cells to fight against the parasites by producing inflammatory cytokines, leading to activation of other immune cells and helping B cells to produce antibodies [46]. Although Tregs and Th17 cells are derived from the same T cell precursors, their function in malaria is the other way around. T cell precursors can be induced to generate Th17 via interleukin 6 and TGF-β, both of which participate in controlling extracellular bacteria and fungi [47], while T cell precursors can be induced to generate Tregs via TGF-β alone, which participates in regulating the immune response [48]. In the present study, we also detected dynamic changes in TGF-β production in *P.y*17XL-infected BALB/c mice (Fig. 8a). Our results indicate that *P.y*17XL infection induced the production of TGF-β and activated the TGF-β/Smad2/3 signaling pathway, which were the primary ways that they regulated the balance of the immune response in the host (Fig. 8b).

If parasites want to enter the host and exist persistently, protein–protein interactions between parasite and host are connected [49]. At present, valuable information from genome, transcriptome and promotion studies have improved our understanding of host–parasite interactions and how parasites evade immune system attack. Parasite-induced pathology is the final result in the complex interactions between host and parasites, involving the host’s genetic constitution, parasite strains, co-infection and environmental features [42, 50]. Over the course of long-term co-evolution, parasites are able to employ complex communication strategies to command or even hijack the host immune system, which can result in parasites having control of physiological and immune homeostasis, which is beneficial to their survival in the host [51]. However, abnormal expression of lncRNAs can result in cell defects or induce growth malformation [52], carcinogenesis [53] and auto-immune diseases in the host [54]. In the present study, we found a number of abnormally expressed lncRNAs that were potentially associated with the imbalance of the immune response in malaria. *Plasmodium* infection was demonstrated to notably alter the expression of lncRNAs in the host. LncRNAs are a bridge to mediate communication between the parasite population and the host immune system.

Research on lncRNAs in the field of immunology is an emerging field of research. The limited number of studies conducted to data have found that lncRNAs, as a type of novel moderator, participate in regulating gene expression in the immune system. LncRNAs regulate the characteristics of immune cell function and differentiation via a highly lineage-specific manner to direct actions with chromatin, RNA and proteins [55]. Consistent with these previous findings, in this study we also found that *Plasmodium* changed the expression of lncRNAs in host cells, resulting in differential gene expression associated with the immune system. In follow-up studies, we need to validate the function of specific lncRNAs that regulate genetic expression related to immunology. Therefore, we emphasize critical gaps in the findings of the present study, and future investigations on the roles of lncRNAs in the immune system and infectious disease are warranted.

## Data Availability

The datasets generated during and/or analyzed during the current study are available from the corresponding author on reasonable request.

## Abbreviations

DCs: Dendritic cells
GOGene: Gene Ontology web server
KEGG: Kyoto Encyclopedia of Genes and Genomes
lncRNAs: Long non-coding RNAs
*P.y*17XL: *Plasmodium yeolii* 17XL
RNA-seq: RNA sequencing
rRNA: Ribosomal RNA
Tregs: T regulatory cells
